# Reliability evaluation oriented dominant dynamic characterization of park-level integrated energy system

**DOI:** 10.1371/journal.pone.0338013

**Published:** 2026-02-06

**Authors:** Jiangang Lu, RuiFeng Zhao, Qian Li, Tian Lan, Hao Wu, Ruilai Xin, Ying Wu, Kai Hou

**Affiliations:** 1 Electric Power Dispatch Control Center, Guangdong Power Grid Co., Ltd., Guangzhou, China; 2 State Key Laboratory of Smart Power Distribution Equipment and System, Tianjin University, Tianjin, China; 3 Tianjin University of Technology, Maritime College, Tianjin, China; Universitas Mercatorum, ITALY

## Abstract

Reliability evaluation of park-level integrated energy systems (PIES) requires capturing the complex interactions among electricity, gas, thermal, and building subsystems, which exhibit typical multiscale dynamic behaviors. Although existing studies have partially considered the dynamic characteristics of PIES, the critical transient responses and the long-term impacts of heat supply interruptions on human health are overlooked. To address these gaps, this paper proposes a reliability evaluation method based on quasi-steady-state simulation. The dynamic behaviors of the electricity, gas, and heating systems, as well as buildings, are analyzed to develop simplified quasi-steady-state models for both energy demand under normal conditions and indoor temperature evolution under extreme scenarios. A new reliability index is then formulated by incorporating the thermal inertia of district heating networks and the temperature tolerance of occupants, forming a comprehensive framework for PIES reliability assessment. Case studies verify that the proposed method enhances the accuracy and interpretability of reliability evaluations, bridging the gap in user health considerations under extreme conditions. The results further reveal that building thermal dynamics play a dominant role in determining the overall reliability performance of PIES.

## 1 Introduction

Amid global climate change, renewable energy integration has accelerated worldwide to reduce carbon emissions and support sustainable development. However, the growing frequency and intensity of extreme weather events such as cold and heat waves, pose unprecedented challenges to the secure and reliable operation of energy infrastructures [1]. For example, the 2021 Texas winter storm caused severe energy shortages and resulted in 111 deaths, revealing limitations in the design of current energy supply systems. Therefore, these events highlight the urgent need to incorporate low-probability but high-impact scenarios into reliability assessment frameworks.

A park-level integrated energy system (PIES) refers to a geographically bounded area featuring integrated energy planning and a certain degree of operational independence. Such systems typically comprise electricity, gas, heating, and cooling subsystems, making them representative models of multi-energy interaction [2–4]. Recent studies have further extended the concept of multi-energy integration to renewable-based configurations, such as offshore wind–floating photovoltaic–hydrogen systems [5], building-integrated photovoltaic/thermal systems for combined electricity and heat generation [6], floating photovoltaic systems coupled with advanced artificial intelligence forecasting frameworks [7], and large-scale solar linear Fresnel reflector systems for green heat and power production [8]. These studies collectively highlight that multi-energy complementarity and coordinated operation can effectively enhance energy utilization efficiency and reliability while supporting sustainable development goals. For instance, by enabling multi-energy complementarity, such as the mutual backup between gas-fired boilers and electric boilers, the reliability of the heating supply in the park can be improved [9]. However, the interconnection among multiple energy carriers also introduces strong interdependencies. For example, a fault in the gas subsystem can propagate to the electricity and heating networks through combined heat and power (CHP) coupling [10]. Reference [11] proposed a reliability evaluation method that considers the cascading features of power and gas systems, while [12] developed a method for electricity–heat PIES under the coupling of electric heat pumps. Reference [13] further introduced a reliability model incorporating multi-energy coordination mechanisms.

However, the dynamic processes within PIES span multiple time scales. The electrical, gas, heating, and hydraulic dynamics typically occur over milliseconds to minutes, while the thermal and building dynamics are governed by the transport of working fluids through pipelines, with temperatures requiring several hours to stabilize. These temporal disparities lead to distinct energy supply characteristics and have a pronounced impact on the manifestation of energy shortages under fault conditions [14]. Studies such as [15–17] incorporated the thermal inertia of buildings into reliability assessments. Reference [18] established a heating load reliability model based on the terminal medium temperature. However, both [15] and [18] neglected the delay induced by thermal transport within district heating networks. To further characterize the impact of heat transfer delays, Reference [19] analyzed the multiscale dynamics of PIES and introduced thermal transfer dynamics into the reliability evaluation, significantly improving model accuracy. Similarly, Reference [20] described the heat transfer dynamics in regional integrated energy systems from the source-network-load perspective, yet both [19] and [20] only accounted for transport delays under mass-based regulation, failing to capture flow-dependent thermal dynamics. Reference [21] employed finite difference methods to discretize the partial differential equations governing transient heat flows into linear equality constraints, thereby establishing a precise electricity–heat PIES model for minute-level energy flow simulations. However, this approach results in computational complexity when applied to large-scale state space analyses required for reliability evaluation.

Since PIES operate with complex energy coupling and mutual dependencies, conventional reliability indices derived from single-energy systems are insufficient to capture the comprehensive performance. Reference [22] proposed an enhanced reliability improvement rate index to reflect the contributions of energy storage and demand response. In terms of thermal load inertia, [16] refined time-dependent reliability indices, while [17] and [19] proposed comfort-based heating reliability metrics accounting for real-time indoor temperature variations. Nevertheless, none of these studies evaluated the health risks associated with prolonged exposure to low-temperature environments.

In summary, previous studies have partially addressed energy transfer delays and thermal inertia but have not fully captured the dynamic behaviors associated with both mass- and flow-based thermal regulation, nor adequately considered the health impacts of prolonged exposure to temperatures beyond human safety thresholds [20]. Existing research on the reliability evaluation of PIES has primarily concentrated on multi-energy coupling mechanisms and energy complementarity, whereas the effects of heat network delays and occupant comfort have received limited attention:

- Most studies neglect the influence of transmission delays in district heating networks and the thermal inertia of buildings on supply reliability. Consequently, the dynamic evolution of indoor temperature during fault occurrence and recovery is often overlooked;
- Current reliability evaluations remain predominantly supply-oriented, paying limited attention to thermal comfort and, more importantly, to human health and safety considerations.

To address these limitations, this study investigates PIES and extends the traditional thermal comfort constraint to a broader human safety perspective. A quasi-steady-state analysis model is developed to represent the multiscale interactions among electricity, gas, and heating subsystems, as well as building thermal dynamics. Based on this framework, a novel reliability evaluation index is proposed to quantify system performance under extreme conditions and to identify the dominant dynamic factors affecting reliability degradation. The main contributions of this paper are summarized as follows:

- Develops a quasi-steady-state analysis model to capture the multiscale dynamic characteristics of PIES;
- Integrates human comfort constraints into the reliability evaluation and systematically investigates the influence of thermal dynamics on assessment outcomes.

## 2 Quasi-steady-state model of PIES

This section constructs the PIES framework and systematically characterizes the coupling mechanisms among electricity, gas, heating, and building subsystems. A quasi-steady-state model is subsequently developed to incorporate transmission delays and building thermal inertia, enabling a more accurate representation of system dynamics.

### 2.1 Standard model of PIES

A typical PIES is generally composed of power, gas, and heating systems, with coupling units include combined heat and power plants, electric boilers, etc [23]. The standard model of PIES can be described as:


$$\left\{ \begin{array}{c} {\dot{x}}_{e}=f_{e}(x_{e},y_{e},u_{e},u_{eh})\\ {\dot{x}}_{g}=f_{g}(x_{g},y_{g},u_{g},u_{eh})\\ {\dot{x}}_{h}=f_{h}(x_{h},y_{h},u_{h},u_{eh})\\ {\dot{x}}_{b}=f_{b}(x_{b},y_{b},u_{b}) \end{array}\right.$$ (1)

where $x_{e}$, $x_{g}$, $x_{h}$, $x_{b}$ denote the state variables of the power, gas, heating, and building systems, respectively. $f_{e}$(•), $f_{g}$(•), $f_{h}$(•), $f_{b}$(•) represent the dynamic models of the power, gas, heating, and building systems, respectively. $y_{e}$, $y_{g}$, $y_{h}$, $y_{b}$ represent the algebraic variables of the power, gas, heating, and building systems, respectively. $u_{e}$, $u_{g}$, $u_{h}$, $u_{b}$ represent the control variables of the power, gas, heating, and building systems, respectively, and $u_{eh}$ represents the control variable of the coupling unit within the energy hub. The control variables can be determined from the state and output variables according to the specified control rates.

### 2.2 Quasi-steady-state model of PIES

As previously discussed, when a fault occurs in the power or gas system, the energy supply to end users is disrupted almost immediately. In contrast, heating and building systems benefit from the thermal inertia of buildings and the stored thermal energy in hot water or steam within the heating network, allowing them to continue providing heating services for a limited period after a fault. During this period, heat supply may be insufficient, causing indoor temperatures to gradually decline toward ambient levels, and recovering once heating service is restored. Due to the thermal dynamics and the delay in heat transfer across the pipeline network, the temperature variation trends among different buildings will differ depending on their respective transmission distances.

In the dynamic analysis of the heating system, the maximum transmission delay $\tau_{h}^{max}$ in the heat energy supply path can be expressed as:


$$\tau_{h}^{\max}=\underset{k\in [1,n_{nh}]}{\max}\left\{\sum_{j\in \Xi_{k}}\frac{l_{j}A_{j}}{m_{j}}\right\}$$ (2)

where $n_{nh}$ represents the number of heat network nodes; $l_{j}$, $A_{j}$, $m_{j}$ denote the pipe length, diameter, flow rate of path j, respectively. $\Xi_{k}$ denotes the set of the kth pathway.

To emphasize the delay effect in the heating system, this section further separates the heating system model into hydraulic and thermal components operating on different time scales. When a fault occurs, indoor temperature remains at their pre-fault values until the working fluid at varying temperatures travels through the heating pipelines to the building. This characteristic of the PIES can be described by the following quasi-steady-state model:


$$\left\{ \begin{array}{c} 0=f_{e}(x_{e},y_{e},u_{e},u_{eh})\\ 0=f_{g}(x_{g},y_{g},u_{g},u_{eh})\\ 0=f_{hh}(x_{h},y_{h},u_{h},u_{eh})\\ {\dot{x}}_{ht}=f_{ht}(x_{ht},x_{ht}^{\tau },y_{h},u_{h},u_{eh})\\ {\dot{x}}_{b}=f_{b}(x_{b},y_{b},u_{b}) \end{array}\right.$$ (3)

where $f_{hh}$(•) and $f_{ht}$(•) represent the hydraulic and thermal subsystem models of the park heating system, respectively. $x_{ht}$ and $x_{ht}^{\tau }$ represent the thermal dynamic variable and the transmission delay dynamic variable of the heating system, respectively. Ignoring the heat absorbed by the pipe wall, the transmission delay effect of the pipeline can be approximated as follows:


$$T_{ab}(x)=T_{a}e^{\frac{-\lambda x}{c_{w}{\dot{m}}_{ab}}}+T_{env}(1-e^{\frac{-\lambda x}{c_{w}{\dot{m}}_{ab}}})$$ (4)

where $T_{ab}(x)$ represents the change in water temperature along the pipeline section *ab* starting from node *a*. $T_{a}$ represents the inlet temperature at node *a*. $T_{env}$ represents the ambient temperature. *x* represents the distance from any cross-section in the pipeline *ab* to the inlet. *x* represents the mass flow rate in the pipeline section *ab* before the fault. *λ* represents the heat transfer coefficient. $c_{w}$ represents the specific heat capacity of water.

**Fig 1:**
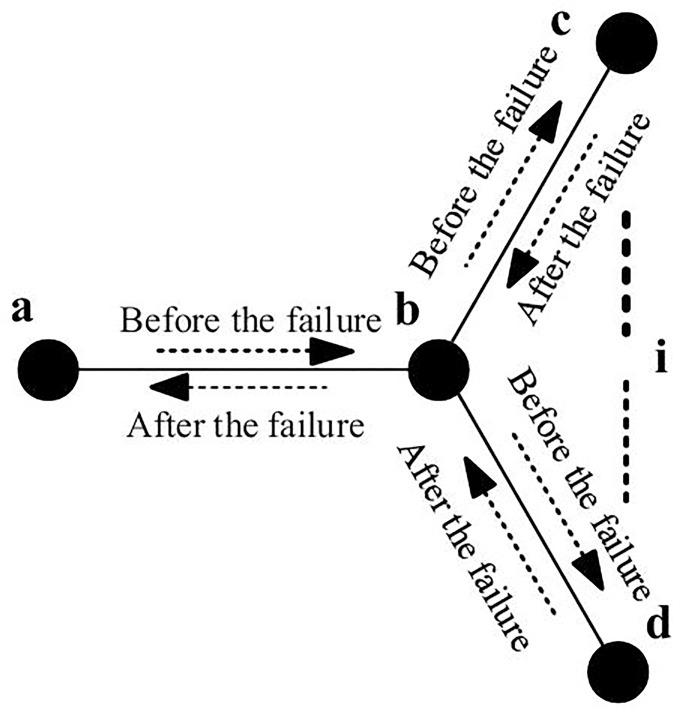
Flow variation of a multi-pipe heating system before and after contingencies.

**Fig 2:**
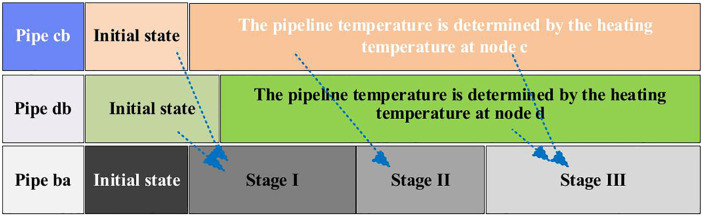
Schematic diagram of the multi-pipe co-simulation approach for quasi-steady state simulation of district heating systems.

Following an emergency, the water flow velocity in the pipeline quickly stabilizes at a new steady state, causing varying degrees of heat loss along the pipeline. In addition, changes in the water temperature of the heat source or other pipelines can alter the inlet temperature of the pipeline. In the three-pipeline simplified system in Fig 1, the outlet temperature of the pipelines undergoes a multi-stage transition before reaching the post-fault steady state depicted. The different initial states at different stages in the collaborative simulation method for pipe *c-b*, pipe *d-b*, and pipe *b-a* are determined, as shown in Fig 2.

In the first stage, $t\in \left[t_{\text{0}},t_{\text{1}}\right]$, the post-fault temperature $T_{a}^{\prime }(t)$ of node *a* is primarily determined by the water temperature in the pipeline section *ab* before the fault and the post-fault flow velocity $m_{ab}^{\prime }$ in the pipeline *ab*. At this time, *x* in formula (4) can be expressed as $x=\frac{{\dot{m}}_{ab}^{\prime }}{A\rho }t$. *A* represents the cross-sectional area of the pipeline *ab*. ρ represents the density of water. In the second stage, $t\in [t_{\text{1}},\infty )$, $T_{a}^{\prime }(t)$ is mainly influenced by the pre-fault temperature of node *b* and the flow velocity entering node *b* after the fault.

The temperature change process of node *a* is as follows:


$$\begin{array}{c} {T^{\prime }}_{a}(t)=h(T_{a},T_{b},{{\dot{m}}^{\prime }}_{ab},t)\\ =\{\begin{array}{c} {}*20c\left(T_{ab}(\frac{{{\dot{m}}^{\prime }}_{ab}}{A\rho }t)-T_{env}\right)e^{-\frac{\lambda \frac{{{\dot{m}}^{\prime }}_{ab}}{A\rho }t}{c_{w}{{\dot{m}}^{\prime }}_{ab}}}+T_{env},t\in \left[t_{0},t_{1}\right]\\ \left({T^{\prime }}_{b}(t)-T_{env}\right)e^{-\frac{\lambda L_{ab}}{c_{w}{{\dot{m}}^{\prime }}_{ab}}}+T_{env},t\in \left[t_{1},\infty \right) \end{array} \end{array}$$ (5)

where $L_{ab}$ denotes the length of the pipeline *ab*, $t_{\text{1}}=t_{\text{0}}+\frac{L_{ab}A\rho }{{\dot{m}}_{ab}^{\prime }}$, h(•) represents the pipeline model, $T_{b}$ represents the pre-fault temperature of node *b*, and $T_{b}^{\prime }(t)$ represents the post-fault temperature of node *b*.

For a node *b* with multiple-source injection, the thermal dynamic processes of different pipelines overlap, resulting in a more complex temperature change process at the node. Any temperature change in a connected pipeline, before it reaches node *b*, will influence the temperature at node *b*. The temperature variation process of node *b* can be described as follows:


$$T_{b}^{\prime }(t)=\frac{\sum {\mathrm{\backslash nolimits}}_{i\in \Omega_{b}}{{\dot{m}}^{\prime }}_{ib}h(T_{b},T_{i},{{\dot{m}}^{\prime }}_{ib},t)}{\sum {\mathrm{\backslash nolimits}}_{i\in \Omega_{b}}{{\dot{m}}^{\prime }}_{ib}}$$ (6)

where $T_{b}$ represents the temperature of node *b* before the fault, and $T_{b}^{\prime }(t)$ represents the temperature of node *b* after the fault, $\Omega_{b}$ represents the set of pipelines flowing into node *b*. $T_{i}$ represents the temperature of node *i* before the fault. $m_{ib}^{\prime }$ represents the mass flow rate of the pipeline section *ib* after the fault.

### 2.3 Numerical implementation method

**Fig 3:**
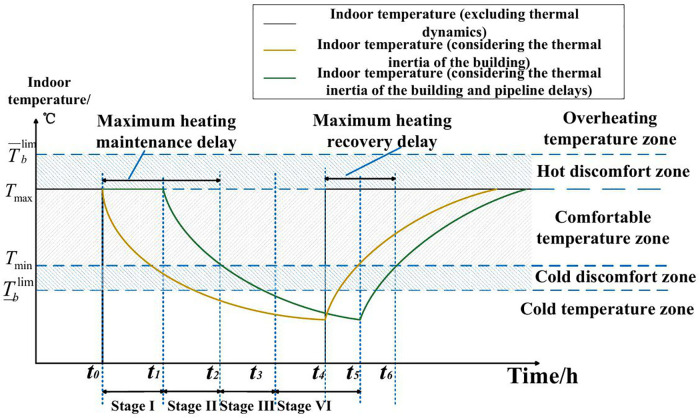
Dynamic behaviors of PIES heat supply after faults.

The simulation process consists of the following steps:

1) Initialization: Based on the multi-energy flow calculation results of the PIES, initialize the temperature and flow velocity throughout the system. The temperature at any pipeline cross-section can be calculated using Equation (4).
2) Post-fault Energy Flow Calculation: Determine the energy flow distribution of the PIES after a fault occurs. This can be achieved using optimal power flow calculations or through pre-defined scheduling rules. The parameters considered include the heat source temperature, mass flow rate of the district heating network, and energy exchange with the power and gas systems.
3) Subsystem Decomposition: Decompose the post-fault dynamic process of the heating system. Given the significant influence of multi-pipeline injection nodes on system dynamics, the heating pipeline network is divided into multiple subsystems using the node incidence matrix. Each subsystem contains either a single heat source or has at most one pipeline beginning at a multi-pipeline injection node. For each subsystem, if the endpoint of a pipeline is a leaf node or a junction of multiple pipelines, the simulation proceeds to the next connected node until all nodes have been processed.
4) Thermal Dynamics Simulation: Simulate the heat transfer process based on the post-fault heat source temperature, network topology, and flow velocity. A multi-pipeline coordinated simulation method is proposed to model overlapping thermal transmission delays on a unified time axis. For pipelines directly connected to the heat source (e.g., *cb* and *db* in Fig 1), the temperature at any location along the pipeline and at the outlet can be obtained using Equations (4) and (5), respectively. For pipelines originating from a multi-source injection node (e.g., *ba*), the inlet thermal dynamics are determined by the injection temperature and flow rate over different time intervals, as described by Equation (6). The downstream heat transfer dynamics in such cases are typically more complex due to source superposition.
5) Health Impact and Heat Loss Evaluation: Evaluate the thermal energy loss and potential health impacts due to inadequate indoor heating. For simplicity, assume that the return temperature of each building is constant. The heating power of a building is computed based on the temperature difference between supply and return water and the corresponding mass flow rate. Fluctuations in heating power affect indoor temperature dynamics, including heating delays during and after the fault. These processes are illustrated in Fig 3.

## 3 Reliability evaluation of PIES considering human comfort

Building on the quasi-steady-state model, human comfort constraints are introduced, and reliability assessment indicators and methods are proposed. By capturing the effects of thermal transmission delays and building thermal storage on temperature dynamics, a comprehensive evaluation framework is established that integrates energy supply with user health and comfort tolerance.

### 3.1 Reliability indicators

Reliability indicators are closely linked to the load losses under various system states. Commonly, the Expected Energy Not Supply (EENS) and load shedding probability are employed to reflect the energy supply reliability of a PIES. Unlike power systems and gas systems, the presence of pipeline transmission delays and building thermal inertia makes calculating load losses in heating systems more complex.

Fig 3 illustrates the indoor temperature response of a heating system load node during an energy supply interruption. When a heating failure occurs at time *t*_*0*_, the user experiences two stages before energy use is affected. In the first stage (*t*_*0*_ to *t*_*1*_), the hot water stored in the pipeline continuously supplies heat to the building, and the indoor temperature of the user is not affected during this stage. In the second stage (*t*_*1*_ to *t*_*2*_), heating becomes insufficient, but due to the thermal inertia of the building, indoor temperature still remains within the human comfort range. The effects of heating losses in these two stages can thus be neglected in reliability assessments. In the third stage (*t*_*2*_ to *t*_*3*_), indoor temperature falls below the minimum comfort threshold, entering a cold discomfort zone, and heat load loss begins to accumulate. Prolonged faults may further reduce temperatures into a super-cold zone, the fourth stage (*t*_*3*_ to *t*_*5*_) shown in Fig 3, posing potential health and life risks to temperature-sensitive occupants. During this stage, the system’s reliability level declines sharply.

Similarly, when analyzing the park-level cooling system, the temperature response curve may shift from a post-fault decrease to a post-fault increase. As the fault persists, indoor temperatures can enter the heat discomfort zone and potentially the overheating zone, possibly reaching the human tolerance limit. To account for the nonlinear effects of these temperature variations on occupants, this paper uses the following piece-wise function for approximation to support the applicability of the description of heating losses in the reliability analysis.



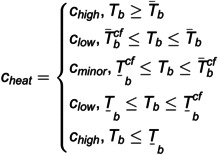

(7)


where *c*_*heat*_ is the piece-wise heat loss coefficient, ${\underset{―}{T}}_{b}^{cf}$ and ${\bar{T}}_{b}^{cf}$ represent the minimum and maximum values of the building comfort temperature, respectively. $T_{b}$ represents the building temperature. ${\bar{T}}_{b}$ and ${\underset{―}{T}}_{b}$represent the lower and upper safety boundaries of the building suitable for human living, respectively. $c_{minor}$, $c_{low}$, and $c_{high}$ represent the heat loss weights under different impacts on the reliability of the park-level integrated energy supply, respectively.

Drawing on the EENS index in power system reliability analysis [24], this paper develops a reliability evaluation index system for regional integrated energy systems, incorporating user thermal comfort at both the node and system levels. First, the node-level reliability evaluation index of the regional integrated energy system is defined as follows:

① Node Load Curtailment Probability ${\text{PLC}}_{j}^{i}$

$${PLC}_{j}^{i}=\sum_{k=1}^{N_{k}}D_{jk}^{i}/TD$$ (8)

where *I* denotes the type of the regional integrated energy system. E、G、H is the type of the regional integrated energy system, and *i*∊{E, G, H}; *TD* is the total evaluation period. For power and natural gas systems. *N*_*k*_ represents the total number of system states in which the *j*-th node of the *i*-th type of energy has a load curtailment during the total evaluation period. For the thermal system, *N*_*k*_ corresponds to the total number of system states in which the indoor temperature at node *j* falls outside the user’s thermal comfort range, specifically when the PMV value at node *j* is below −0.5 during the duration of a certain system state. For power and natural gas systems, $D_{jk}^{i}\;$denotes the duration of the failure state or sub-state *k* affecting the load point *j* of the *i*-th type of energy. For the thermal system, *D i jk* is the duration of the failure state or sub-state *k* in which the load point *j* does not meet the user’s thermal comfort.

② System Expected Load Curtailment Frequency ${\text{EFLC}}_{j}^{i}$

$${EFLC}_{j}^{i}=\frac{8760}{TD}{NF}_{j}^{i}$$ (9)

where for power and natural gas systems, NF*i j* represents the number of times the load point *j* of the *i*-th energy type transitions from a load-curtailment state to a non-curtailment state. For the thermal system, NF*i j* denotes the number of times the load point *j* transitions from a state that does not meet thermal comfort to one that satisfies it. It should be noted that if, under a certain system state, the load point *j* of the thermal system fails to fully meet the user’s thermal comfort during the duration of this state, then the load point *j* is regarded as being in a state that does not meet the thermal comfort in this system state.

③ Average Duration of Node Load Curtailment ${\text{ADLC}}_{j}^{i}$

$${ADLC}_{j}^{i}=\frac{8760\cdot {PLC}_{j}^{i}}{{EFLC}_{j}^{i}}$$ (10)

④ Expected Energy Service Not Supplied

The EESNS*i j* index is used to quantify the reliability of the PIES. Different from EENS, the EESNS evaluation considers the impact of low-temperature conditions on the human body and corrects the load curtailment amount based on the quasi-steady-state model of the PIES:


$${EESNS}_{j}^{i}=\left\{ \begin{array}{c} \frac{8760}{TD}\sum_{k=1}^{N_{k}}P_{k}^{i}C_{k}^{i}D_{k}^{i},i\in E,G\\ \frac{8760}{TD}\sum_{k=1}^{N_{k}}\sum_{j}c_{heat}\int_{0}^{t_{jk}}\left(L_{jk}^{i}-\Phi_{jk}^{i}(t)\right),i\in H \end{array}\right.$$ (11)

where P(S) represents the probability of the occurrence of system state *S*. $n_{s}$ represents the number of faulty components in system state *S*. n represents the total number of components in the system. $U_{i}$ represents the unavailability rate of component *i* in system state *S*. $S_{x}$ represents the set of system states with load curtailment. C(S) represents the load curtailment amount in system state *S*. For power and natural gas systems, $C_{jk}^{i}$is the load curtailment amount of the load point k of the *i*-th type of energy in system state *k*, and *D*_*k*_ is the duration of the corresponding system state. For the thermal system, *c*_heat_ is the piece-wise heat loss coefficient, and its definition and value-taking method are shown in Equations (3–15). $L_{jk}^{i}$ is the average load (MW) of the load point *j* of the thermal system in system state *k*. $\Phi_{jk}^{i}$(*t*) is the input power (MW) of the load point *j* of the thermal system at time *t* in system state *k*, and *t*_*jk*_ is the time when the load point *j* of the thermal system does not meet *t*he user’s thermal comfort in system state *k*.

The heat load losses $C(S{)}_{p}$ and $C{\left(S\right)}_{b}$ caused by the transmission delay of the heating pipeline and the heat-storage characteristics of the building can be calculated by the following formula:


$$C(S{)}_{p}=\sum \Phi_{i}-\frac{\int_{0}^{t_{fault}}c_{w}m_{q}^{i}(T_{s}^{i}-T_{o}^{i})dt}{t_{fault}}$$ (12)

$$C(S{)}_{b}=\frac{\int_{0}^{t_{fault}}R_{b}(T_{b}^{bd}-T_{b})sgn(T_{b}^{bd}-T_{b})c_{heat}dt}{t_{fault}}$$ (13)

where $\Phi_{i}$ represents the heat load of node *i*. $t_{fault}$ represents the fault duration. $c_{w}$ represents the specific heat capacity of water. $m_{q}^{i}$ represents the net mass flow rate injected into node *i*. $T_{s}^{i}$ and $T_{o}^{i}$ represent the supply water temperature and outlet temperature of node *i*, respectively. $R_{b}$ represents the heat loss coefficient of the building. $T_{b}^{bd}$ represents the lower limit of the building heat load loss. sgn(·) is the sign function. When z>0, sgn(z)=1; when z≤0, sgn(z)=0.

### 3.2 Reliability evaluation method of PIES

This paper uses the state-enumeration method to evaluate the reliability of PIES. The specific process is as follows:

**Step 1**. Input the PIES configuration data, component failure and repair rates, power/gas/heat demand profiles, and load-curtailment cost parameters.

**Step 2.** Assume all equipment is operating normally. Use the energy-hub model together with steady-state power–gas–heat network models to solve the steady-state flows and obtain initial operating conditions for the coupled system.

**Step 3.** Enumerate system states from the PIES topology and component failure/repair data to generate the set of possible fault states.

**Fig 4:**
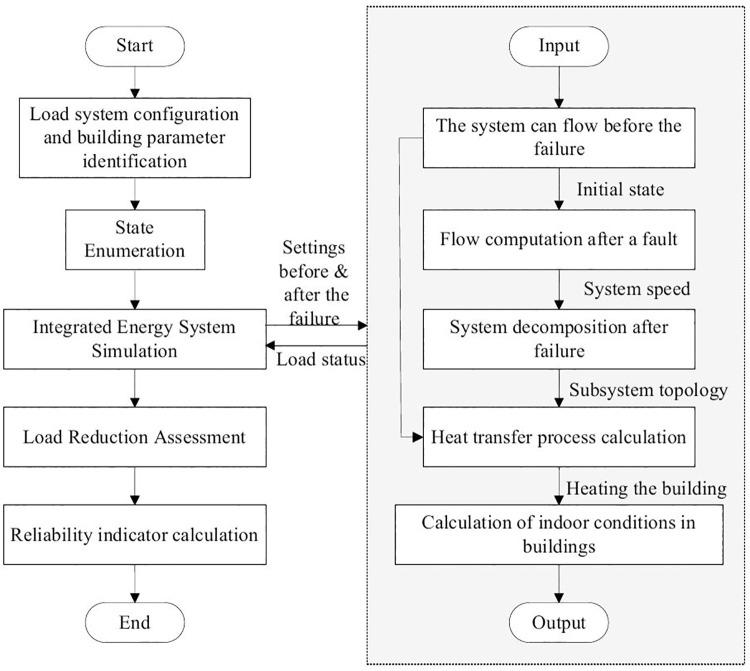
Flowchart of PIES reliability evaluation.

**Step 4.** For each heating-system fault state, run the quasi-steady-state simulation module (right side of Fig 4) using the pre- and post-fault conditions to obtain the corresponding power–gas–heat system states and the heating-system/building temperature trajectories.

**Step 5.** Simulate pipeline heat-transmission dynamics using post-fault heat-source temperatures, network topology, and flow velocities. Apply a multi-pipeline coordinated simulation to model overlapping transmission delays.

**Step 6.** Quantify thermal energy loss and potential health impacts from insufficient indoor heating. Assume each building’s return temperature is constant; compute delivered heating power from the supply–return temperature difference and the mass flow rate.

**Step 7.** Using human comfort and safety constraints, construct the piecewise weighting function for generalized load loss. Then apply Equations (10) and (11) to compute the equivalent load loss that combines energy shortfall with occupants’ temperature tolerance.

**Step 8.** Combine fault probabilities with the calculated load losses and use Equations (8) and (9) to compute the reliability indices, namely the expected energy service not supplied and related metrics.

## 4 Case study

In this section, a coupled model that integrates building dynamics and human tolerance is established to validate the effectiveness of the quasi-steady-state simulation-based reliability evaluation method for PIES. The simulations are performed on a hardware platform equipped with an Intel i7-9700HQ 3.00 GHz CPU and 16 GB RAM.

### 4.1 Basic data

**Fig 5:**
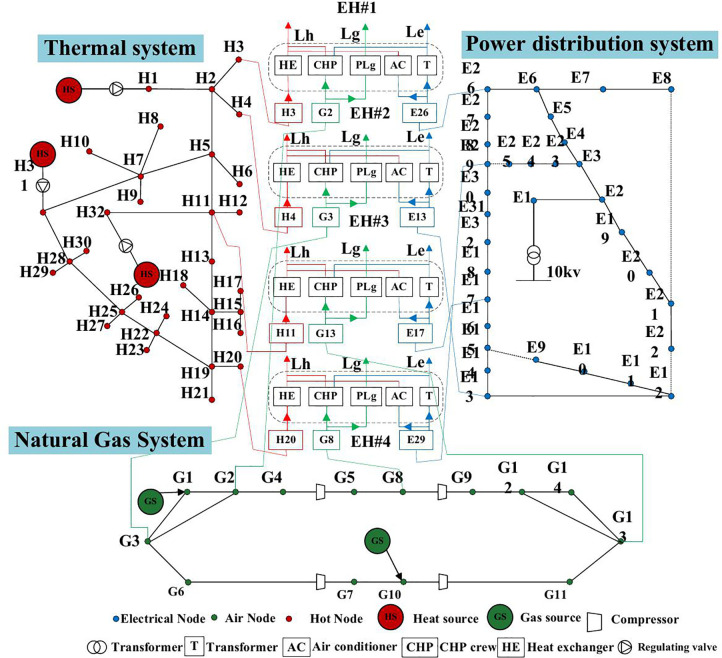
Schematic diagram of the studied PIES.

This section employs the PIES illustrated in Fig 5 to simulate the operational behavior of an industrial park energy supply system, thereby validating the effectiveness of the proposed methodology. The system comprises a 30-node power grid, a 14-node gas network, a 32-node thermal network, and 21 buildings, with multi-energy networks interconnected via four energy hubs. Detailed system parameters are provided in [25]. The case study focuses on evaluating the impact of system failures on building heating, which can result in excessively low indoor temperatures. The normal thermal comfort range for building interiors is defined as 18°C to 24°C, with a minimum acceptable temperature of 9°C during prolonged outages [26]. The ambient temperature is assumed to be 0°C. System failure rates, repair times, and load loss costs for reliability analysis are adopted from [27].

### 4.2 Result analysis

#### 4.2.1 Building parameter identification.

**Fig 6:**
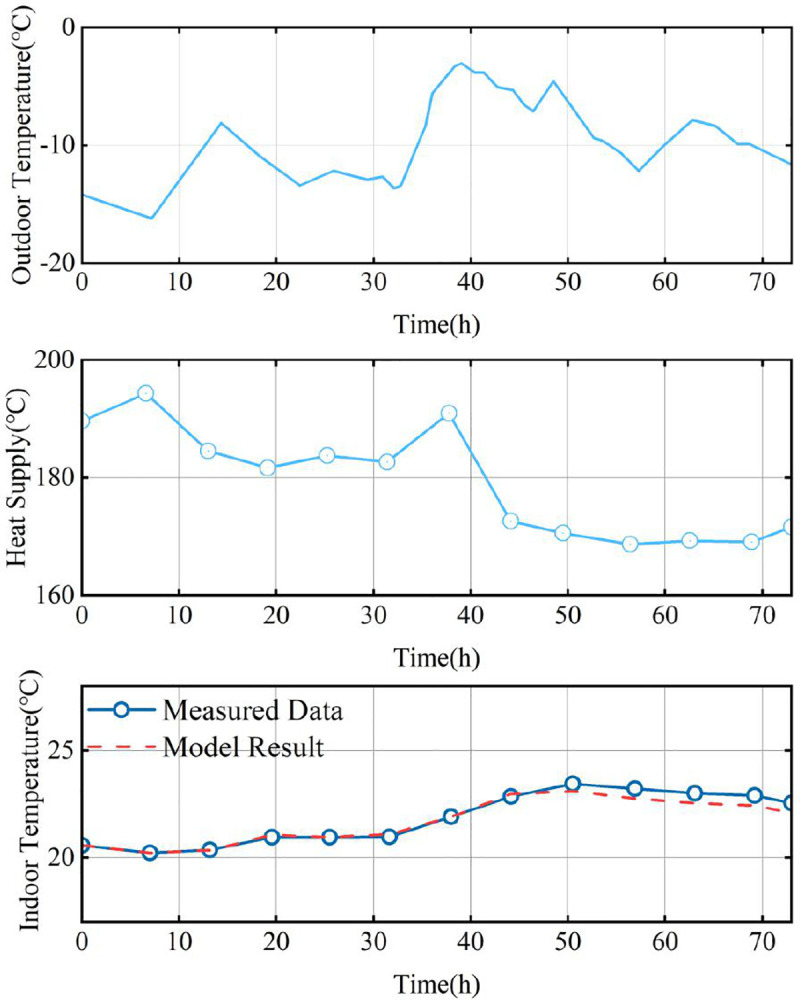
Building parameters extracted from field data.

In the proposed reliability analysis method considering indoor temperature characteristics, building parameters are crucial for accurate evaluation. To verify the feasibility of extracting equivalent building parameters from practical system data, Fig 6 demonstrates the modeling of building-injected heating power alongside averaged indoor temperature measurements under specified external temperature variations. These measurements are derived from actual heat exchange station data using the building model in [28]. The results show that the identified model accurately captures indoor temperature dynamics, thereby supporting reliability analysis requirements for PIES.

#### 4.2.2 Impact of transmission delay on heating power.

**Fig 7:**
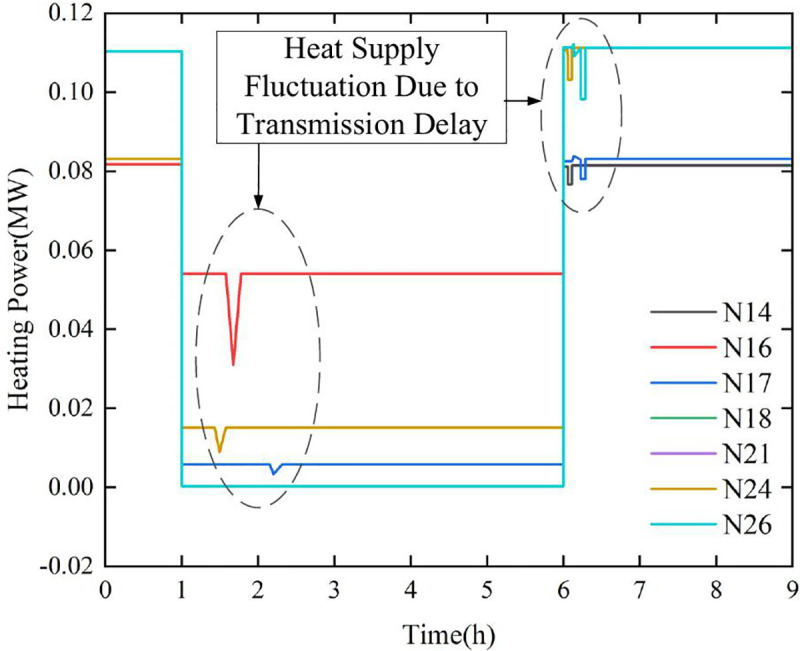
Impact of transport delay on the heat supply of PIES.

To illustrate the effects of pipeline transmission delays and building thermal dynamics, a fault scenario in pipes 13–14 of the heating system is analyzed, as shown in Fig 5. The fault occurs at the start of the first hour, prompting the dispatch system to adjust the thermal network’s mass flow rate based on steady-state optimal power flow results to minimize thermal losses. The fault is cleared at the sixth hour, after which the mass flow rate returns to its pre-fault level, as illustrated in Fig 7.

The results reveal multiple abrupt power fluctuations at nodes due to transmission delays during both fault occurrence and recovery phases. While these fluctuations may impact control system performance, the delayed temperature decline induced by transmission delays leads to a gradual temperature recovery once supply is restored. Consequently, these transient power variations temporally smooth temperature transitions without significantly compromising heating service quality.

#### 4.2.3 Impact of thermal dynamics on PIES reliability.

This section evaluates system performance under three scenarios: single-energy mode, multi-energy interconnected mode, and multi-energy interconnected mode with thermal dynamics considered. It compares the reliability of the integrated energy system across different configurations and examines how varying thermal dynamics affect the evaluation results:

Case 1: Independent operation of the power, gas, and thermal subsystems, excluding inter-system coupling, quasi-steady-state transmission behavior of the thermal system, and building thermal inertia [29].

Case 2: Coupled operation of the power, gas, and thermal subsystems, while neglecting the quasi-steady-state transmission behavior of the thermal system and building thermal inertia [30].

Case 3 (Proposed Method): Coupled operation of the power, gas, and thermal subsystems, incorporating both building thermal inertia and thermal transmission delays. A quasi-steady-state simulation method is used to evaluate system reliability under fault conditions.

As shown in Table 1, compared with traditional single-energy supply modes, the proposed RIES model achieves improved reliability indices (PLC, EFLC, EESNS) across all subsystems, demonstrating that multi-energy complementarity enhances both energy utilization efficiency and supply reliability. The proposed method exhibits notable advantages over existing approaches: while Case 1 highlights single-energy limitations and Case 2 emphasizes multi-energy complementarity, the proposed Case 3 further incorporates thermal dynamics and comfort-related constraints, thus revealing hidden risks and reflecting user-oriented reliability more accurately.

**Table 1:** Reliability indices.

Case	PLC			EFLC (occurrences/year)			ADLC(hours/year)			EESNS(MWh/year)		
	Power	Gas	Thermal	Power	Gas	Thermal	Power	Gas	Thermal	Power	Gas	Thermal
**Case1**	0.0279	0.1487	0.0199	16.0811	81.5563	11.5579	15.9927	15.9821	15.0955	3.8157	15.6422	12.7473
**Csae2**	0.0120	0.0285	0.0106	6.5302	17.6351	6.4286	15.2192	14.1696	14.4596	1.4945	1.2941	1.6016
**Case3**			0.0185			5.8533			24.3059			6.2596

From the perspective of thermal comfort, incorporating slow thermal dynamics in Case 3 results in higher PLC and ADLC indices but a reduced EFLC for the thermal subsystem compared with Case 2. This outcome results from the redefinition of thermal reliability criteria, shifting the emphasis from simple energy deficiency to indoor temperature acceptability. During transient heating shortages, indoor temperatures decline gradually rather than instantaneously; hence, thermal comfort requirements may still be satisfied, and the impact of load curtailment becomes apparent only after the shortage persists. Furthermore, due to thermal inertia, temperature recovery following fault clearance may extend across multiple system states.

The increase in EESNS observed in Case 3 results from the introduction of segmented thermal loss coefficients (C_heat). Prolonged heating shortages lower indoor temperatures below acceptable thresholds, which in turn lengthens the recovery period. This prolonged exposure to low temperatures not only heightens health risks but also leads to greater economic penalties, thereby increasing both C_heat and EESNS. These results confirm that the quasi-steady-state model provides a more realistic and interpretable reliability evaluation by explicitly capturing heat network delays and user comfort dynamics.

#### 4.2.4 Node-level reliability analysis considering thermal dynamics and user tolerance.

**Fig 8:**
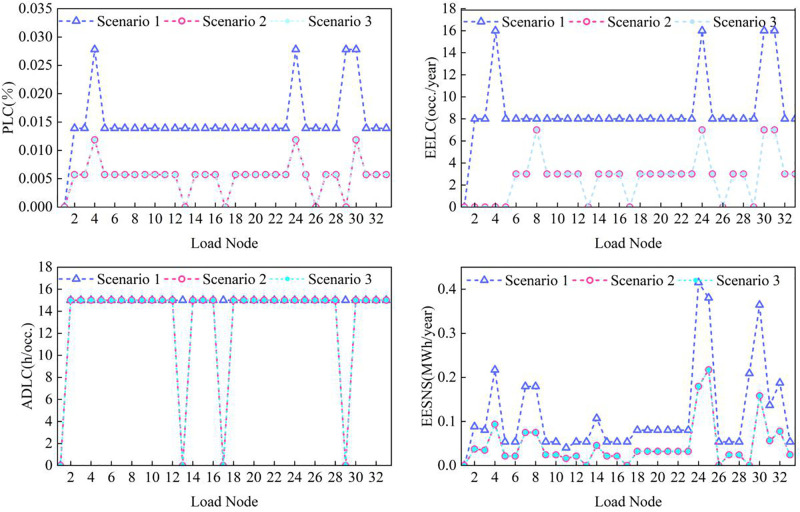
Reliability indicators of power system load nodes under different cases.

**Fig 9:**
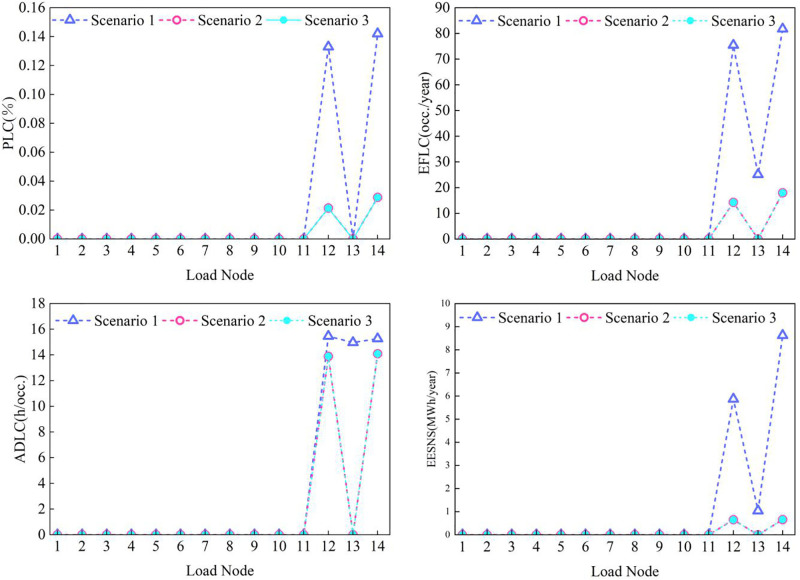
Reliability indicators of gas system load nodes under different cases.

**Fig 10:**
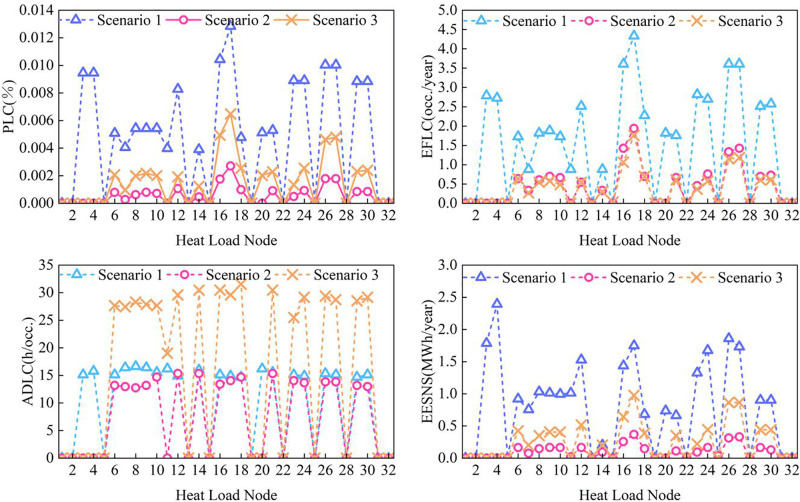
Reliability indicators of heat system load nodes under different cases.

Node-level reliability indices for power, gas, and thermal subsystems are evaluated using the methodology in Section 3.2.3. Node-level analysis is critical for identifying system vulnerabilities and assessing the impact of coupling devices and the proposed reliability framework. The results for each subsystem are presented in Figs 8–10.

Analysis of Fig 8 reveals that reliability indices of most electrical load nodes exhibit convergence, which can be attributed to the power subsystem’s single input node (E1) connected to the distribution grid through one transmission line. A fault in this line leads to system-wide load curtailment. Consequently, power supply reliability can be enhanced by introducing redundant feeders or distributed backup sources.

Compared with Case 1, all power nodes show markedly improved reliability in the energy-coupled cases (Cases 2 and 3). Nodes E13, E17, E26, and E29—each connected to coupling devices—exhibit particularly significant improvements. This enhancement arises because coupling devices enable other subsystems to offset power shortages by increasing their own outputs, thereby reducing dependence on the electrical network.

Overall, this node-level reliability analysis confirms that the spatial distribution of reliability aligns with the proposed dominant dynamic characterization, highlighting that building thermal inertia exerts a leading influence on the reliability response of the PIES.

Fig 9 reveals that gas nodes G12 and G14 exhibit lower reliability compared with other nodes. This can be attributed to the ring topology of the gas network—faults in the pipelines connected to these nodes increase their effective distance from gas sources, making them more susceptible to load curtailment under supply-demand imbalances. The integration of coupling devices significantly enhances reliability across all gas nodes, with node G13 (connected to a coupling device) showing the most notable improvement. This demonstrates that deploying coupling devices at critical load points can effectively enhance gas supply reliability.

Overall, this node-level reliability analysis confirms that the spatial distribution of reliability aligns with the proposed dominant dynamic characterization, underscoring that building thermal inertia remains the dominant factor governing the reliability response of the PIES.

For the thermal subsystem shown in Fig 10, the radial topology causes branch nodes to become isolated and experience load shedding when pipeline faults occur, whereas mainline nodes (e.g., H7 and H14) maintain higher reliability. In energy-coupled cases, the reliability of thermal nodes improves significantly—particularly at nodes H3, H4, H11, and H20, which are connected to coupling devices. This improvement arises because coupling devices enable other subsystems to compensate for thermal shortages by adjusting their outputs. Therefore, strategically deploying coupling devices at vulnerable thermal nodes can effectively enhance heating supply reliability.

However, under Case 3, thermal node reliability slightly declines compared with the conventional coupled cases. This is primarily due to the integration of multi-energy coupling and multi-timescale dynamics into the reliability assessment. Even if thermal load curtailment does not occur in a given state, residual effects from previous states—such as indoor temperatures falling below comfort thresholds—can reduce reliability indices in subsequent states. These results indicate that the proposed quasi-steady-state reliability assessment framework more accurately captures real user-side energy consumption dynamics and better represents actual system reliability performance.

Overall, the spatial distribution of reliability remains consistent with the proposed dominant dynamic characterization, confirming that building thermal inertia serves as the primary factor influencing the reliability response of the PIES.

## 5 Conclusion

This study develops a quasi-steady-state PIES model for reliability analysis of energy supply systems, extending traditional thermal deficiency assessments to account for building thermal comfort. The results show that heating systems’ thermal inertia and transmission delays enable temporary service continuity after heat source failures, while mass flow regulation can mitigate such failures, highlighting the importance of considering electro-hydraulic-thermal interactions. Segmented thermal deficiency metrics, based on human comfort, safety, and hazardous thresholds, improve reliability evaluation accuracy compared to conventional methods.

In addition, the proposed quasi-steady-state framework involves several simplifications. Transient thermal–hydraulic dynamics in district heating networks are linearized, and uncertainties in renewable generation are not explicitly captured. Moreover, the analysis assumes fixed building parameters and typical weather conditions, which may limit its applicability under highly variable or extreme environments. Future research will incorporate stochastic modeling, energy storage, and demand-side management to address these limitations and further enhance the robustness of the proposed reliability evaluation framework.
